# Changes of protein expression during tumorosphere formation of small cell lung cancer circulating tumor cells

**DOI:** 10.32604/or.2022.027281

**Published:** 2023-03-01

**Authors:** SANDRA STICKLER, BARBARA RATH, MAXIMILIAN HOCHMAIR, CLEMENS LANG, LUKAS WEIGL, GERHARD HAMILTON

**Affiliations:** 1 Institute of Pharmacology, Medical University of Vienna, Vienna, Austria; 2 Karl Landsteiner Institute of Lung Research and Pulmonary Oncology, Klinik Floridsdorf, Vienna, Austria; 3 Department of Trauma Surgery, Sozialmedizinisches Zentrum Ost, Donauspital, Vienna, Austria; 4 Division of Special Anesthesia and Pain Medicine, Medical University of Vienna, Vienna, Austria

**Keywords:** SCLC, Spheroid, Chemoresistance, EpCAM, Topotecan, Epirubicin

## Abstract

Small cell lung cancer (SCLC) is frequently disseminated and has a dismal prognosis with survival times of approximately two years. This cancer responds well to initial chemotherapy but recurs within a short time as a globally chemoresistant tumor. Circulating tumor cells (CTCs) are held responsible for metastasis, the extremely high numbers of these cells in advanced SCLC allowed us to establish several permanent CTC cell lines. These CTCs are distinguished by the spontaneous formation of large spheroids, termed tumorospheres, in regular tissue culture. These contain quiescent and hypoxic cells in their interior and are associated with high chemoresistance compared to single cell cultures. Nine CTC lines were compared for their expression of 84 proteins associated with cancer either as single cells or in the form of tumorospheres in Western blot arrays. With the exception of the UHGc5 line, all other CTC lines express EpCAM and lack a complete EpCAM-negative, vimentin-positive epithelial-mesenchymal transition (EMT) phenotype. Upon formation of tumorospheres the expression of EpCAM, that mediates cell-cell adhesion is markedly upregulated. Proteins such as E-Cadherin, p27 KIP1, Progranulin, BXclx, Galectin-3, and Survivin showed variable changes for the distinct CTC cell lines. In conclusion, EpCAM presents the most critical marker for individual SCLC CTCs and the assembly of highly chemoresistant tumorospheres.

## Introduction

Small cell lung cancer (SCLC) is a neuroendocrine lung tumor representing 15% of lung cancers and shows metastases in most patients at first presentation [1,2]. Smokers with heavy tobacco consumption for decades have a high risk of developing SCLC [3]. SCLC shows an inactivation of tumor suppressor genes p53 and retinoblastoma (Rb1), as well as an increased expression of a wide range of diverse oncogenic drivers expressed in distinct subpopulations of patients [1,4]. Standard care for disseminated SCLC is platinum-based chemotherapy and prophylactic cranial irradiation (PCI) [5,6]. Recurrences within 1–2 years affecting the liver, brain, bone, and other secondary sites follow initially high responses. As second-line therapies, the single approved chemotherapeutic Topotecan or anthracycline-based regimens yield low and short-lived responses [5,6]. Treatment has not changed for the last decades because the evaluation of all novel drugs, targeted and anti-angiogenic agents failed to provide superior survival over standard chemotherapy [5–7].

The typical overall 5-year survival of 5–10% for extended SCLC requires new treatment modalities and characterization of the mechanism of drug resistance. The dissemination of SCLC seems to be related to the excessively high counts of circulating tumor cells (CTCs) compared to other malignancies [8,9]. A minority of these CTCs have tumor-initiating properties [10]. Our lab has successfully established nine SCLC CTCs *in vitro* that grow continuously in tissue culture [11,12]. These SCLC CTC lines derived from distinct patients exhibit similar expression of proteins, cytokines, and various receptors, indicating their essential role in metastatic SCLC. Access to the SCLC CTC lines offers a unique opportunity to study the metastatic potential and chemoresistance of the SCLC CTCs.

**Figure 1:**
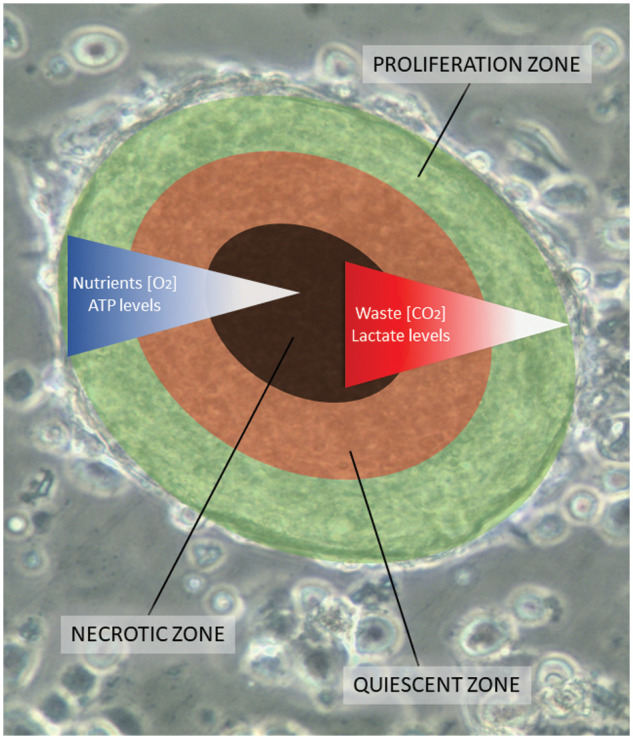
A scheme of a tumorosphere overlying an actual cluster light microscopic picture. Tumorospheres have been analyzed in a previous work by immunohistochemistry demonstrating KI-67-positive proliferation zones and hypoxic CA9-positive core [12]. The inserted bar corresponds to a length of 100 µm. The proliferation zone of the tumorosphere is shown in green color, the quiescent zone in orange, and the core necrotic zone in black, respectively.

All SCLC CTC lines exhibit the spontaneous formation of large multicellular aggregates under normal cell culture conditions, which grow to diameters of 1–2 mm, designated tumorospheres [11,12]. Normally, tumor spheroids only form in response to culture conditions that prevent attachment of the cells, such as hanging drop cultures or use of low-adherence substrates and other methods [13,14]. Tumorospheres grown to large sizes show a lack of nutrients and oxygen, and an accumulation of waste products in their interior [15,16]. 3D cultures or spheroids exhibit increased resistance to chemotherapeutics and irradiation due to the presence of quiescent cells, cell contact-mediated effects, and lack of generation of oxygen radicals in their cores [17–21]. Fig. 1 shows an overlay of the different zones with an underlying tumorosphere. These spheroids contain an outer proliferative zone adjacent to a quiescent zone and a necrotic core, indicating a hypoxic region and accumulation of cellular waste products.

Cancer cells grown *in vitro* as spheroids represent exactly the chemoresistance phenotype of native solid tumors and display pathways of resistance linked to hypoxia, altered chromatin structure, impairment of apoptosis, cell cycle alterations, and decreased drug perfusion [22]. Ki-67-expressing cells are enriched in the outer layers, quiescent cells near the core and, expression of carbonic anhydrase IX (CAIX) in the inner regions indicates at least transient hypoxia [23,24]. Comparative cytotoxicity tests employing four common drugs used in the therapy of SCLC reveal significantly increased chemoresistance of all tumorospheres. This may account for the observed clinical refractoriness of relapsed patients. In general, the expression of numerous genes differs between cells growing as multicellular tumor spheroids (MCTS) compared to monolayer cultures. Cell lines derived from tumors or normal tissues cultivated in form of 2D cultures exhibited altered expression of genes in up to 70% of cases compared to 3D cultures [25]. MCTSs closely resemble gene expression profiles of *in vivo* growing tumors [15,16,26]. The chemoresistance of ovarian cancer spheroids could be entirely reversed by the dissociation of the spheroids [27].

Despite high investments to develop new cancer therapeutics, about 90% of drugs finally tested in clinical trials fail [28,29]. Spheroids with sizes of 250–750 μm are detectable in cancer patients, and correspondingly, traditional testing methods are increasingly being complemented by 3D models of human tumors. Relapsed SCLC are highly chemo resistant most likely to the impaired penetration of diverse cytotoxic drugs into tumorospheres. Chemo resistant spheroids were demonstrated to differentially express genes involved in an extracellular matrix organization, and cell adhesion but their dissociated single cells are chemosensitive [25,27]. In conclusion, chemoresistance of CTCs from relapsing SCLC patients seem to depend on the protein interactome involved in cell aggregation. Such interaction of several expressed proteins is difficult to be deduced from transcriptomics data. Universal chemoresistance due to the formation of large clusters may not be restricted to SCLC but seems to occur in glioblastoma, neuroectodermal tumors and others. Effective clinical strategies to overcome this physical barrier are currently under investigation. Tumorosphere-related drug resistance emulates the clinical picture of the putative cancer stem cell (CSC)-associated resistance, but CSC-targeting therapy has not translated to successful clinical treatments so far [30].

The mechanisms leading to the spontaneous formation of SCLC CTC tumorospheres are not clear, and therefore, we searched for differences in protein expression between single cells and corresponding spheroids using Western blot arrays. The Oncology XL array assesses the relative expression levels of 84 human cancer-related proteins comprising growth factor receptors, signaling mediators, growth factors, adhesion proteins, proteases, chemokines, hormones, and others. The CTCs in form of single cells or tumorospheres were checked for their chemosensitivity using Topotecan and Epirubicin, which are administered for second-line treatment of SCLC. These spheroids contain quiescent and hypoxic cells and seem to constitute a vital component of the global drug resistance of SCLC tumors.

## Materials and Methods

### Patients and cell lines

The SCLC CTC cell lines BHGc7, 10, 16, 26, 27, 71, 500, 590, and UHGc5 were established from blood samples of extensive disease-SCLC patients following disease progression under second-line therapy at our institution as described previously [11]. Blood collection and generation of cell lines was done according to the Ethics Approval 366/2003 by the Ethics Committee of the Medical University of Vienna, Vienna, Austria, using EDTA blood vacuum tubes. Cells were finally cultured in RPMI-1640 medium (Seromed, Berlin, Germany) supplemented with 10% fetal bovine serum (Seromed) and antibiotics (Penicillin-Streptomycin; Sigma-Aldrich, St. Louis, MO, USA). CTCs proliferate in the form of single cells, and tumorospheres in parallel, and the spheres were collected by sedimentation or with the help of 100 µm cell strainers (Corning, Corning, NY, USA).

### Cytotoxicity assays

1 × 10^4^ cells in the form of single cells or tumorospheres (TOS) in 100 μl medium were distributed to wells of 96-well microtiter plates (TPP, Trasadingen Switzerland). Therafter, ten 2-fold dilutions of the test compounds were added from stock dilutions. Assays were at least performed in triplicate. Calculating CTC cell size and the surface area of the globular tumorospheres, 1 × 10^4^ suspension cells correspond to the same number of cells accessible at the surface of the spheroids. The mean size of the clusters used for the cytotoxicity tests was determined, and the number of clusters/well adjusted accordingly. The plates were incubated for four days under tissue culture conditions, and viable cells were detected using a modified MTT assay (EZ4U, Biomedica, Vienna, Austria). The respective dilutions of the compounds tested are present for the whole incubation period. IC_50_ values were determined from dose-response curves using Origin Pro, Version 9.9 (OriginLab Corporation, Northampton, MA, USA).

### Protein expression arrays

We determined the relative expression levels of 84 cancer-related proteins in the investigated cell lines by performing a Proteome Profiler Human XL Oncology Array (R&D Systems (Catalog # ARY026)). The kit consists of 4 nitrocellulose membranes spotted in duplicate with antibodies for cancer-related proteins. Membranes were treated following the enclosed protocol (Protocol for Multiple Analyte Detection using the Proteome Profiler™ Human XL Oncology Array Kit, Panel A (Catalog # ARY026)). Cell culture lysates of 9 cell lines in the form of single cells and their tumorospheres were obtained by removing particulates in samples by centrifugation. Afterward, samples were diluted and incubated with the Human XL Oncology Array overnight, followed by removal of unbound proteins by washing. Subsequently, the array was set with a detection antibody cocktail, followed by applying Streptavidin-HRP and chemiluminescent detection reagents. These reagents produce a signal corresponding to the amount of protein-bound, which was visualized using a ChemiDoc™ Touch Imaging System (Bio-Rad Laboratories Inc., California, USA).

### Heatmaps

Heatmaps were plotted for nine cell lines in the form of single cells and their tumorospheres using protein expression data of 43 cancer related proteins in the application heat map with dendrogram in Origin Version 9.9 (OriginLab Corporation, Northampton, MA, USA). Thereby a hierarchical cluster analysis was performed. Firstly, clustering rows of 43 genes by group average and Pearson correlation to reveal differences in gene expression via z-score of these genes in 9 cell lines in the form of single cells and their tumorospheres. Secondly, to show the differences in expression of these proteins via z-score within tumorospheres and uncover similarities between the different cell lines by clustering rows and columns by group average and Pearson correlation. Missing values were filled up with column means, while standardization was performed for columns.

### Reactome pathway database analysis

The Reactome database (Version 3.7) organizes signaling and metabolic molecules and their relations into biological pathways and processes [31]. The respective proteins were subjected to Reactome analysis, and the most overrepresented pathways were recorded, including their statistical significance.

### Statistical analysis

Analysis of the data from cytotoxicity assays and heatmap analyses were calculated using the Origin 9.9 software, in particular, the heatmap option with dendrograms. A *p*-value < 0.05 was regarded as statistically significant. The significance of the heatmap data (single cells *versus* tumorospheres) is presented in Suppl. Data 1, with substantial differences coded as number 1 and nonsignificant differences as number 0.

## Results

### Chemosensitivity of the SCLC CTC lines as single cells and tumorospheres

**Figure 2:**
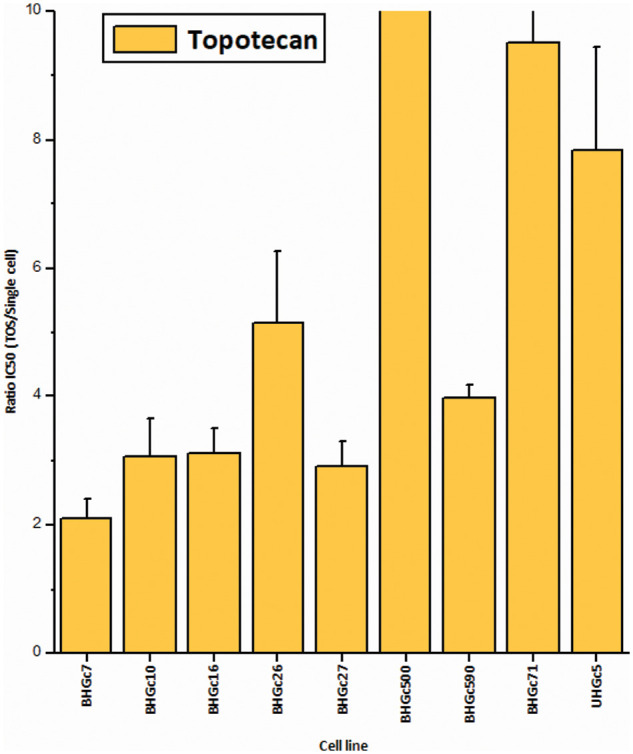
Ratios of Topotecan sensitivity of tumorospheres/single cells (mean values ± SD). IC_50_ values were calculated from dose-response experiments using Topotecan with an initial concentration of 10 µg/ml and 10 twofold concentrations. The Ratios for BHGc7 and BHGc71 are 54.7 ± 17.9 and 23.5 ± 4.1, respectively. All results are statistically significant.

Fig. 2 shows the increase of the chemoresistance to Topotecan of tumorospheres compared to single cells. Single CTC cells and corresponding CTC tumorospheres were exposed to Topotecan for four days and the IC50 values were calculated from the dose-response curves. The IC50 values of the cell lines for single cells are presented in Suppl. Data 2. Dependent on the specific cell line, the spheroids are a factor of two to > ten more resistant to the drug. The ratio of BHGc500 is 15.1 ± 3.4.

**Figure 3:**
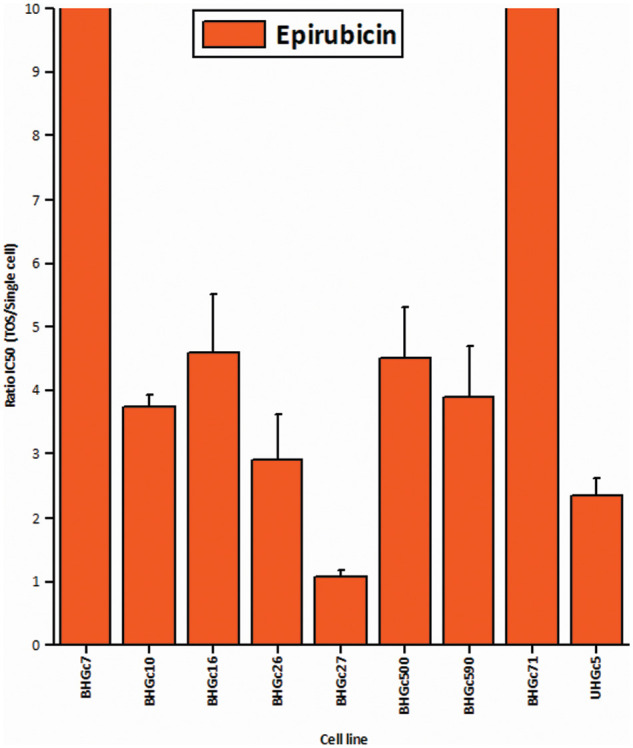
Comparison of the IC50 values of single CTC cells and tumorospheres treated with Epirubicin (mean values ± SD).

Fig. 3 shows the ratios of chemosensitivities of tumorospheres compared to the corresponding single cells. The IC_50_ values of the cell lines for single cells are presented in Suppl. Data 2. Dependent on the specific cell line, the spheroids are a factor of 2.4 to >ten more resistant to the drug, with the exception of BHGc27, which exhibits no significant difference for single cells and tumorospheres. Epirubicin is the most active component of the second-line regimen consisting of Epirubicin, Cyclophosphamide, and Vincristine. The results show that the increase in resistance to Epirubicin in tumorospheres differs from the results obtained with Topotecan (correlation coefficient r^2^ = 0.11).

### Western blot array of oncogene-related proteins for the formation of tumorospheres

**Figure 4:**
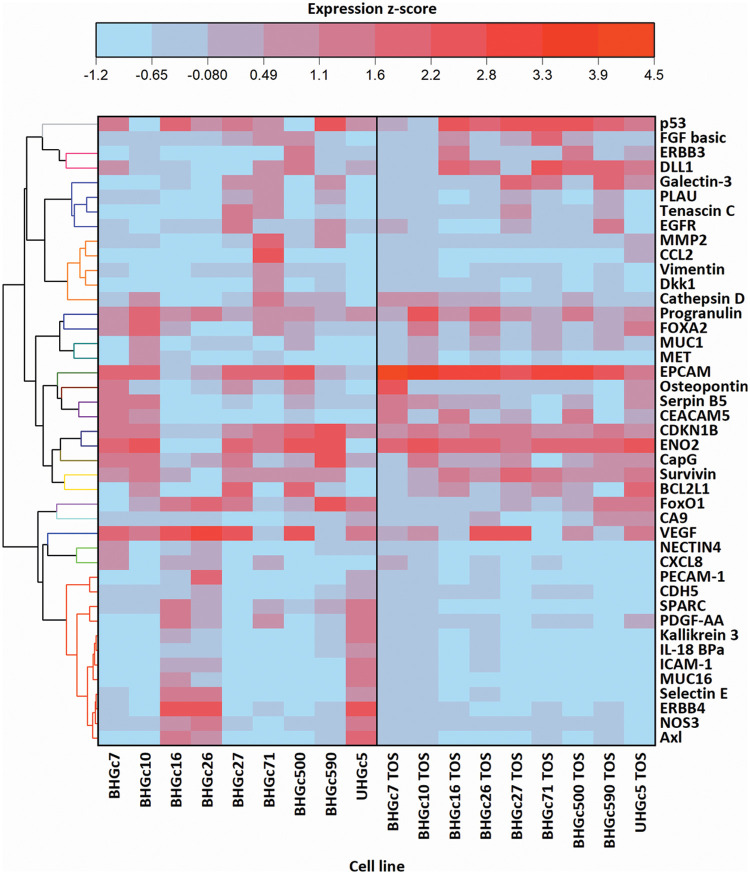
Comparison of 9 cell lines in the form of single cells (left) and tumorospheres (TOS) (right) regarding the expression of 43 of the 85 cancer-related proteins detected by a Proteome Profiler Human XL Oncology Array (R&D Systems) (Catalog # ARY026).

Fig. 4 shows a range of cancer-related proteins that was compared for single SCLC CTC cells, the corresponding tumorospheres and 43 selected proteins that were found to be changed are presented in the heatmap. After normalization, a hierarchical clustering was performed for the heatmap. The most consistent alteration was found to be the upregulation of EpCAM. The statistically significant differences and the direction of the alterations (up- or downregulation) are presented in Suppl. Data 1.

Fig. 4 further shows that BHGC16, BHGc26, and UHGc5 exhibited expression of several proteins that became downregulated in the tumorospheres. All three cell lines have the highest proliferation rates among all SCLC CTCs.

**Figure 5:**
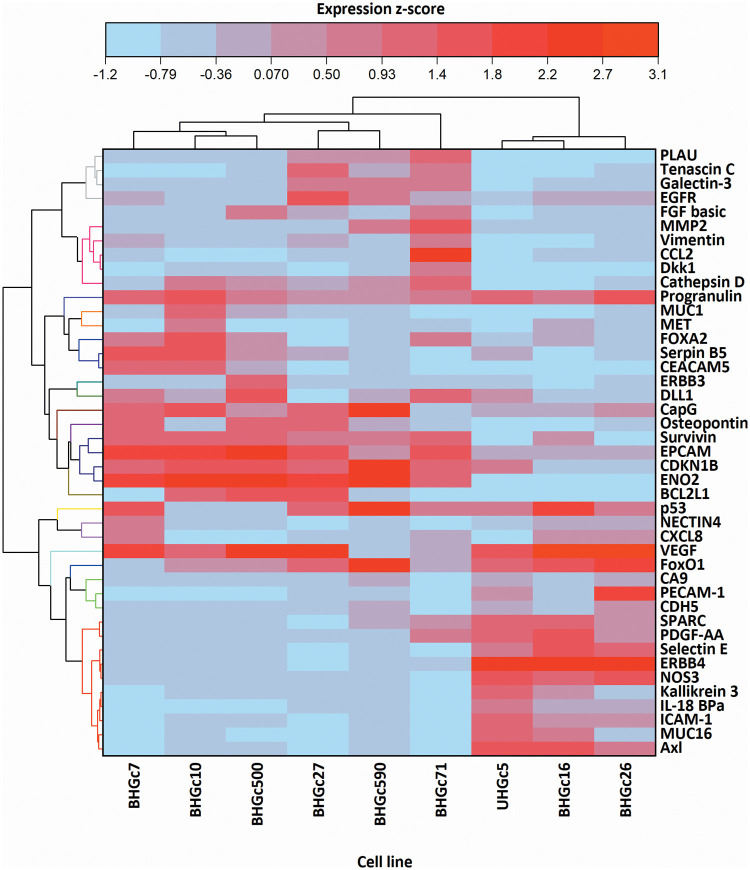
Heatmap comparison of 9 CTC cell lines in the form of single cells and 43 cancer-related proteins detected by a Proteome Profiler Human XL Oncology Array. The heatmap was calculated in Origin Pro, Version 9.9 (OriginLab Corporation, Northampton, MA, USA).

In Fig. 5, where the single cells CTC cells were clustered individually, UHGc5, BHGc16, and BHGc26 showed the closest relationship. While Fig. 4 shows that high expression of 14 cancer-related proteins gets lost during the formation of the corresponding tumorospheres. The comparison of the levels of expression of EpCAM for single CTC cells and the corresponding tumorospheres in Fig. 5 shows a significantly increased expression of the antigen, with the exception of the BHGc27 cells. Statistics evaluation is presented in Suppl. Data 1.

**Figure 6:**
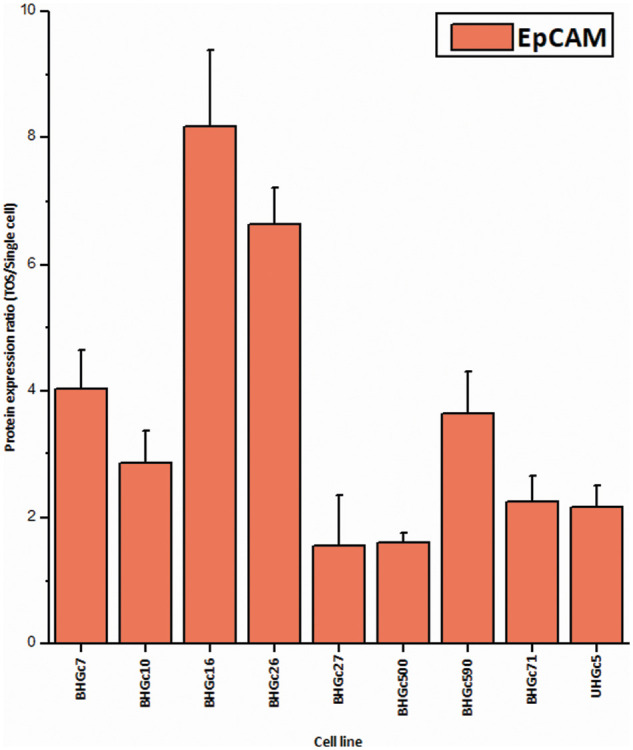
Ratios of EpCAM expression tumorospheres/single cells (mean values ± SD). With exception of BHGc27, the increase in the expression of EpCAM in tumorospheres is significant.

### Reactome pathway analysis

The Reactome pathway analysis of the 14 proteins with reduced expression in tumorospheres (Nectin 4, CXCL8, PECAM-1, CDH5, SPARC, PDGFF-AA, Kallikrein 3, IL-18 BPa, ICAM-1, MUC16, Selectin E, ERBB4, NOS3, Axl) revealed a connection of these genes to signal transduction. In detail, there were several pathways involving ERBB2, e.g., signaling by ERBB2 in cancer (*p* = 3.35E^−5^), downregulation of ERBB2 signaling (*p* = 3.35E^−5^), ERBB2 regulates cell motility (*p* = 5.03E^−6^), signaling by ERBB2 KD mutants (*p* = 3.09E^−5^), signaling by ERBB2 TMD/JMD mutants (*p* = 2E^−5^), ERBB2 activates PTK6 signaling (*p* = 4.28E^−6^), GRB2 events in ERBB2 signaling (*p* = 6.77E^−6^), SHC1 events in ERBB2 signaling (*p* = 3.35E^−5^). Furthermore, pathways involving ERBB4, e.g., signaling by ERBB4 (*p* = 5.29E^−9^), nuclear signaling by ERBB4 (*p* = 1.95E^−10^), SHC1 events in ERBB4 signaling (*p* = 6.77E^−6^).

These 14 proteins further influence the signaling of interleukins, which are known for their importance in the function of the immune system, e.g., Interleukin-10 signaling (*p* = 7.01E^−9^), Interleukin-18 signaling (*p* = 9.84E^−7^), Inteleukin-4 and Interleukin-13 signaling (*p* = 1.33E^−6^). Furthermore, they are associated with Receptor Tyrosine Kinase signaling (*p* = 2.36E^−9^), long-term potentiation (*p* = 2.15E^−5^), PI3K/AKT signaling in cancer (*p* = 5.82E^−5^), and regulation of PI3K/AKT signaling by PI5P, PP2A, and IER3 (*p* = 6.78E^−5^). In summary, these alterations affect cellular signaling, proliferation, and interleukin pathways that are effective in the three CTC cell lines exhibiting the highest proliferative activity.

## Discussion

Disseminated SCLC is universally resistant to second-line treatment regardless of the administration of conventional therapeutics, targeted agents, or diverse novel compounds [32]. In contrast to NSCLC, SCLC has no addiction to specific oncogenic drivers but lacks intact tumor suppressor genes p53 and RB1. Furthermore, SCLC exhibits vigorous growth in response to complex epigenetic alterations. Thus, the therapy of SCLC still relies on cytotoxic drugs that have been in use for the last decades. The global chemoresistance to structurally unrelated drugs has not been correlated to distinct cellular mechanisms of resistance or general refractoriness to apoptosis. The panel of newly established SCLC CTC lines exhibits the spontaneous formation of large spheroids, termed tumorospheres, that ultimately reach diameters of 1–2 mm in tissue culture. As demonstrated previously, these spheroids are comprised of an outer layer of proliferative cells and inner layers of quiescent and hypoxic cells, resulting in a resistance to chemoradiotherapy [12].

These findings point to a cell physiological mechanism of chemoresistance linked to tumorospheres by limited drug perfusion, and lower chemosensitivity of the quiescent and hypoxic cells in their interior. The respective CTC cell lines are tumorigenic in immunocompromised mice (data not shown). 3D cultures of tumor cell lines resulting in the formation of different spheroids have been propagated as models of actual tumors that are more representative than traditional 2D cultures. However, as opposed to SCLC CTCs, this buildup of spheroids has to be enforced by nonadherent tissue culture conditions such as hanging drops, agitation, or various scaffolds. The so-called MCTS closely mimics *in vivo* solid tumors’ features, such as structural organization, gradients of oxygen, pH, and nutrients as well as resistance to chemotherapy and radiotherapy [33,34]. Beyond a size of approximately 500 μm, MCTS represent avascular tumors with a proliferating outer rim, an internal quiescent zone, and a necrotic core due to the lack of nutrients and oxygen [35,36]. MCTSs morphology present as compact spheroids expressing high E-cadherin levels, tight aggregates with overexpression of N-cadherin, or as loose aggregates of cells [37,38]. Compactness and size of the MCTSs control chemoresistance since loosely aggregating cells are sensitive to treatment, whereas compacted spheroids are more resistant, and very large spheroids (>~500 μm diameter) show treatment refractoriness [39,40]. The hypoxic conditions in the core of spheroids, as observed in the interior of tumorospheres, induce hypoxia-inducible factor 1 (HIF-1) and CAIX that are linked to drug resistance [12].

The proteins participating in the spontaneous formation of tumorospheres in SCLC CTCs have not been characterized. Chemosensitivity tests demonstrated again that the tumorospheres are more resistant to Topotecan and Epirubicin compared to the corresponding single-cell suspensions [12]. The CTCs as single cells are highly sensitive to both drugs indicating that the high resistance of SCLC to second-line treatment is most likely not at the cellular level [41]. Here, we have analyzed a panel of 85 cancer-related proteins in Western blot arrays for single CTCs and the corresponding tumorospheres.

For array analysis, the cells and spheroids are lysed, and in the latter case, proteins from proliferative and quiescent cells are mixed in the lysate. Among the proteins analyzed, EpCAM was the single factor that showed consistently higher expression in tumorospheres *versus* single cells. The formation of spheroids by Caco-2, SW480, and HCT116 colorectal adenocarcinoma cell lines under low-adhesion culture conditions were reported to show a 3-fold higher expression of EpCAM compared to monolayers, but no changes in 19 other markers [42]. The proteins most commonly downregulated here comprise growth factor receptors, such as the EGFR/ERB family, cell adhesion proteins, p27KIP1, the chemoattractants CCL2 and CCL8, as well as proteins related to angiogenesis and mesenchymal phenotype. Furthermore, reduced expression of SPARC (Secreted Protein and Rich in Cysteine) regulates the interaction of tumor cells with the extracellular matrix [43]. Cell clusters in balanced dormancy show low proliferation and avascular anti-angiogenic cellular cues [44,45,46]. Large clusters grow up to 1–2 mm without vascularization and display central necrotic cores but can be triggered toward invasive progress in response to factors such as angiogenic signals, inflammatory cytokines, and others [47]. Drugs that suppress HIF-1α expression, such as Topotecan, Irinotecan, or Adriamycin, block the angiogenic outgrowth and prolong dormancy. In conclusion, the anti-angiogenic, anti-proliferative and, anti-inflammatory features of the tumorospheres resemble characteristics of dormant tumor cells [44].

EpCAM is frequently upregulated in primary tumors and metastases, particularly in adenocarcinomas, certain squamous cell carcinomas, and others [48]. Increased EpCAM expression in patients is correlated with poor prognosis and therapeutic refractoriness because EpCAM supports cancer growth and progression through modulation of cell proliferation, differentiation, migration, and invasion [49,50]. However, the reduced expression has been reported for EpCAM and cytokeratin for cancer cells undergoing epithelial-mesenchymal transition (EMT) during metastasis [51]. High expression of EpCAM observed in some CTCs has been ascribed to cells during the mesenchymal-epithelial transition (MET) in the circulation before intravasation [52]. Overexpression of EpCAM on CTCs makes it a promising marker for cancer diagnosis as well as monitoring and isolation of CTCs [53]. In particular, the single approved CTC detection system, namely CellSearch©, relies on EpCAM-mediated immunomagnetic isolation of CTCs. EpCAM function seems to mediate cell adhesion by interaction with other EpCAM molecules on neighboring cells by homophilic interaction [54]. EpCAM participates in the formation of tight junctions and regulates claudin by direct effects [55]. Phenotypic changes in MCTSs were reported to be associated with dysregulated networks, including alterations in claudins, plexin 2, several integrins, syndecans, EPCAM, and E–cadherin with high EpCAM expression linked to larger tumors, metastasis, and inferior survival [56]. Furthermore, EpCAM and β-catenin were reported to be expressed in spheroids derived from hepatocellular carcinoma (HCC) cells [57]. EpCAM-positive HCC cells exhibited improved spheroid, formation in 3D culture and were more tumorigenic and invasive to the lung *in vivo*. Detection of disseminated tumor cells (DTC) in bone marrow (BM) of patients with early-stage NSCLC has been associated with poor outcomes [58]. BM aspirates from 104 non-metastasized NSCLC patients that underwent potentially curative tumor resection revealed CK+ and EpCAM+ DTCs in half of the patients, respectively. EpCAM+, but not CK+ DTCs in BM, predicted reduced PFS and tumor-specific survival [59]. Antibodies to EpCAM have been developed for cancer therapy, either as a native monoclonal agent, anti-EpCAM toxin-conjugated antibody, or IL2-conjugated antibody that exhibit promising clinical activity [60].

In conclusion, the formation of highly chemoresistant tumorospheres by SCLC CTCs is accompanied by elevated expression of EpCAM, a protein involved in cell-cell adhesion. Therefore, EpCAM-positive CTCs seem to be the cell population destined to survive in circulation and lead to chemoresistance and metastasis.

## Figures and Tables

**Supplementary Data 1:**
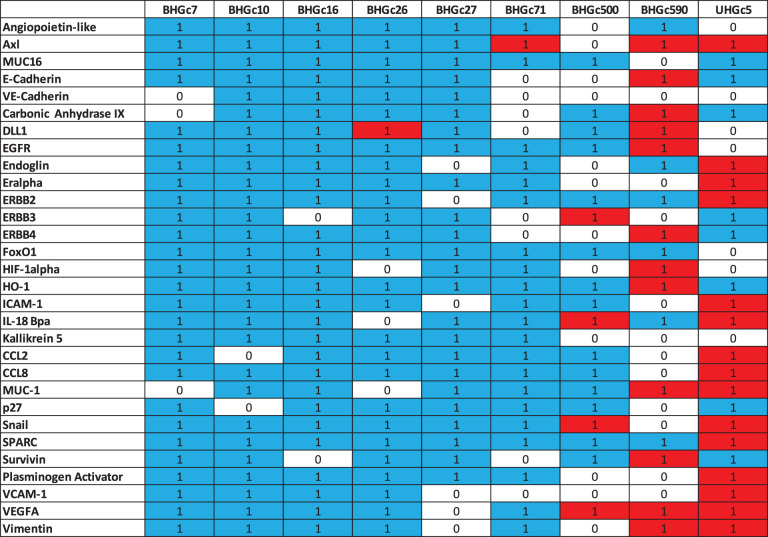
Heat map statistics. Statistically differences in the expression of cancer-related proteins between single cells and tumorospheres are indicated by 1 (0 not significant). Red color indicates an increase and blue color a decrease in expression.

## Data Availability

Data is available upon reasonable request.

## Appendix

Data show that CTCs as single cells are sensitive to both chemotherapeutics.

**Supplementary Data 2:** Table of IC50 values for single cell CTCs and Topotecan/Epirubicin

Cell line	Epirubicin IC50 [µg/ml]	Std	Topotecan IC50 [µg/ml]	Std
**BHGc7**	0.01	0.01	0.62	0.47
**BHGc10**	0.03	0.03	0.98	1.04
**BHGc16**	0.04	0.06	0.27	0.28
**BHGc26**	0.05	0.04	0.12	0.05
**BHGc27**	0.004	0.0003	0.01	0.01
**BHGc500**	0.02	0.01	0.03	0.02
**BHGc590**	0.14	0.07	0.85	0.41
**BHGc71**	0.15	0.05	0.19	0.18
**UHGc5**	0.02	0.03	0.02	0.02
